# Transcriptome Analysis of Methyl Jasmonate-Elicited *Panax ginseng* Adventitious Roots to Discover Putative Ginsenoside Biosynthesis and Transport Genes

**DOI:** 10.3390/ijms16023035

**Published:** 2015-01-29

**Authors:** Hongzhe Cao, Mohammed Nuruzzaman, Hao Xiu, Jingjia Huang, Kunlu Wu, Xianghui Chen, Jijia Li, Li Wang, Ji-Hak Jeong, Sun-Jin Park, Fang Yang, Junli Luo, Zhiyong Luo

**Affiliations:** Molecular Biology Research Center, State Key Laboratory of Medical Genetics, School of Life Sciences, Central South University, Changsha 410078, China; E-Mails: caohongzhe1986@hotmail.com (H.C.); nzaman_global@yahoo.com (M.N.); haoxiu1990@163.com (H.X.); huangjj1985@126.com (J.H.); wukunlu@163.com (K.W.); chenxianghui@163.com (X.C.); lijijia1986@126.com (J.L.); wangli@mail.csu.edu.cn (L.W.); jhjeong@126.com (J.-H.J.); sunjpark@126.com (S.-J.P.); Yangfang2010@csu.edu.cn (F.Y.); jlluo@scripps.edu (J.L.)

**Keywords:** *Panax ginseng*, adventitious root, methyl jasmonate, Illumina/Solexa, transcriptome, ginsenoside biosynthesis, PDR (pleiotropic drug resistance) transporters, MVA (mevalonic acid) pathway

## Abstract

The *Panax ginseng* C.A. Meyer belonging to the Araliaceae has long been used as an herbal medicine. Although public databases are presently available for this family, no methyl jasmonate (MeJA) elicited transcriptomic information was previously reported on this species, with the exception of a few expressed sequence tags (ESTs) using the traditional Sanger method. Here, approximately 53 million clean reads of adventitious root transcriptome were separately filtered via Illumina HiSeq™2000 from two samples treated with MeJA (Pg-MeJA) and equal volumes of solvent, ethanol (Pg-Con). Jointly, a total of 71,095 all-unigenes from both samples were assembled and annotated, and based on sequence similarity search with known proteins, a total of 56,668 unigenes was obtained. Out of these annotated unigenes, 54,920 were assigned to the NCBI non-redundant protein (Nr) database, 35,448 to the Swiss-prot database, 43,051 to gene ontology (GO), and 19,986 to clusters of orthologous groups (COG). Searching in the Kyoto encyclopedia of genes and genomes (KEGG) pathway database indicated that 32,200 unigenes were mapped to 128 KEGG pathways. Moreover, we obtained several genes showing a wide range of expression levels. We also identified a total of 749 ginsenoside biosynthetic enzyme genes and 12 promising pleiotropic drug resistance (*PDR*) genes related to ginsenoside transport.

## 1. Introduction

Ginseng (*P. ginseng* C.A. Meyer) is well recognized as the king of herbs and widely used as a source of herbal medicine in eastern Asia and North America [1]. The genus “panax” means panacea or cure all and the root is so-called “ginseng” because of its man-like shape and thought to represent its three characteristics (body, mind and sprit) [2]. The plant has multiple clinical and pharmacological effects related to cancer, stress, obesity, diabetes and cardiovascular disease [3,4,5,6]. The major pharmacologically active compounds of ginseng are ginsenosides belonging to dammarane and oleanane type triterpene saponins. Dammarane-type consists of two groups based on their structure, *i.e.*, the Rb group (protopanaxadiols, including Ra1, Ra2, Rb1,Rb2, Rb3, Rc, Rd, Rg3, Rh2 and others) and the Rg group (protopanaxatriols, including Rg1, Rg2, Re, Rf, Rh1 and others), while oleanane-type ginsenoside represents only one saponin, Ro [7]. They have been identified in all parts of the plant, including the root, stem, leaf and flower [8]. However, research for years on its highly valued root has largely been conducted due to its important herbal applications since ancient times.

The large size (~3.2 Gb) and high complexity of the *P. ginseng* genome that is reportedly tetraploid (2*n* = 4*x* = 48) [9] has made it difficult to get a whole genomic sequence for this plant. Some researchers have generated ESTs from untreated and MeJA treated ginseng hairy roots by the traditional Sanger sequencing method to detect genes involved in ginsenosides biosynthesis [10,11,12]. Over the last few years, the next-generation sequencing (NGS) technology has emerged as a cutting edge approach for high-throughput sequence determination of model and non-model organisms, especially those with large and complex genomes. Besides, this approach has considerably improved our understanding on the complexity of gene expression profiling, discovery, regulation and networks in plants [13,14]. In spite of its clear potential, the NGS technology has been applied in a few studies for the transcriptome analysis of *Panax* species, including *P. ginseng*, *P. notoginseng* and *P. quinquefolius* [15,16,17], where the technology was almost limited to the Roche/454 pyrosequencing platform to identify mainly ginsenoside biosynthetic genes. More recently, the Illumina/Solexa platform has been introduced in untreated adventitious root transcriptome analysis of two *P. ginseng* cultivars namely Chunpoong (CP) and Cheongsun (CS) for their gene expression profiling and ginsenoside biosynthetic genes identification [14]. However, gene discovery and candidate genes involved in ginsenosides biosynthesis and their transport in ginseng species are still limited.

In recent years, plant genetic engineering research has turned towards modulation of the secondary metabolite biosynthesis pathway, and biotic and abiotic stress and defense-related genes [18,19]. Secondary metabolites biosynthesis and their regulation is a multilayer network requiring common signal pathways and a variety of key elicitors such as MeJA or salicylic acid [20]. Hence, Jasmonic acid and its derivatives have received much attention as key molecules, particularly in regulating the secondary metabolism in many plant species. Increasing evidence shows that MeJA significantly increases the production of many highly valuable secondary metabolites of pharmaceutical and industrial importance [21]. Various secondary metabolites including ginsenosides synthesized by MeJA in medicinal plants have pharmacological effects in human health and play important roles in the adaptation of plants to a particular environment [10]. Among various elicitors, MeJA has recently been confirmed as an effective elicitor for the induction of ginsenoside contents in cultured cells and adventitious roots of *P. ginseng* [22,23]. In addition, MeJA significantly up-regulates *PDR* gene expression related to various secondary metabolites including ginsenosides accumulation and their transport in cells [24,25]. In this study, we adopted the Illumina/Solexa sequencing technology for sequencing and analyzing the MeJA treated adventitious root transcriptome of *P. ginseng* and a total of 71,095 all-unigenes were generated from Pg-Con and Pg-MeJA. After annotation, *P. ginseng* was shown as genetically more related to *Vitis vinifera*. Furthermore, we have conducted the identification of several candidate genes encoding almost all the enzymes associated with ginsenoside biosynthesis via the mevalonic acid (MVA) pathway. We have also discovered a number of *PDR* genes related to the transport of ginsenosides in cells. To the best of our knowledge, this is the first MeJA elicited NGS transcriptome sequencing and analysis of *P. ginseng* adventitious roots that will supply very useful resources for further functional research on this plant and its closely related species.

## 2. Results and Discussion

### 2.1. Results

#### 2.1.1. Sequencing Output and Transcriptome Assembly

We obtained a total of 55,166,532 and 54,784,414 raw reads from Pg-Con and Pg-MeJA respectively. By filtering all the raw reads, we obtained 52,821,238 (95.75%) clean reads from Pg-Con and 52,661,812 (96.13%) from Pg-MeJA with an average length of 90 bp. High-quality reads from two samples were combined and provided to the transcriptome assembly program “Trinity” [26]. A total of 136,776 and 132,474 contigs were assembled with an average length of 316 and 307 bp and N50 of 525 and 448 bp from Pg-Con and Pg-MeJA respectively. Later, contigs were further assembled into 75,956 unigenes with a mean size of 698 bp from Pg-Con and 73,439 with a mean size of 690 bp from Pg-MeJA. There were 33,672 (≥500 bp) and 17,543 (≥1000 bp) unigenes in Pg-Con, while 32,393 (≥500 bp) and 16,598 (≥1000 bp) unigenes in Pg-MeJA. The majority of unigenes were smaller than 500 bp in both cases that accounted for approximately 56% of all unigenes. Finally, the unigenes from both samples were assembled into 71,095 all-unigenes in total, with a mean length of 871 bp and N50 of 1307. An overview of assembled sequences has been summarized in Table 1.

**Table 1:** An overview of assembled sequences produced by “Trinity” following Illumina/Solexa sequencing.

Length of Nucleotides (bp)	Contigs		Unigenes		All-unigenes
	Pg-Con	Pg-MeJA	Pg-Con	Pg-MeJA	
0–199	85,621	82,631	14,443	12,401	0
200–299	16,761	17,177	12,719	13,661	13,923
300–399	9576	9502	9213	9269	9976
400–499	5430	5063	5909	5715	6472
500–599	3330	3359	4428	4400	4916
600–699	2581	2429	3568	3622	4180
700–799	1897	1806	3039	2882	3459
800–899	1611	1538	2738	2608	3089
900–999	1351	1270	2356	2283	2789
1000–1099	1129	1049	2081	2033	2476
1100–1199	969	910	1899	1870	2321
1200–1299	864	761	1642	1582	2006
1300–1399	753	670	1572	1435	1862
1400–1499	686	641	1387	1276	1731
1500–1599	593	544	1179	1218	1558
1600–1699	519	482	1111	1083	1441
1700–1799	496	384	997	932	1273
1800–1899	395	383	905	875	1189
1900–1999	322	285	688	639	920
2000–2099	257	234	597	537	748
2100–2199	248	193	481	425	638
2200–2299	194	159	478	340	571
2300–2399	177	154	357	306	482
2400–2499	119	109	255	296	407
2500–2599	120	96	272	237	374
2600–2699	108	99	239	223	311
2700–2799	88	64	184	154	242
2800–2899	81	56	173	133	223
2900–2999	56	57	125	133	192
≥3000	444	369	921	871	1326
Total Number	136,776	132,474	75,956	73,439	71,095
Total length (bp)	43,209,082	40,612,192	52,997,248	50,685,207	61,942,217
Mean length (bp)	316	307	698	690	871
N50 (bp)	525	488	1161	1130	1307

#### 2.1.2. Functional Annotation of Predicted Proteins in Nr and Swiss-Prot Databases

**Figure 1:**
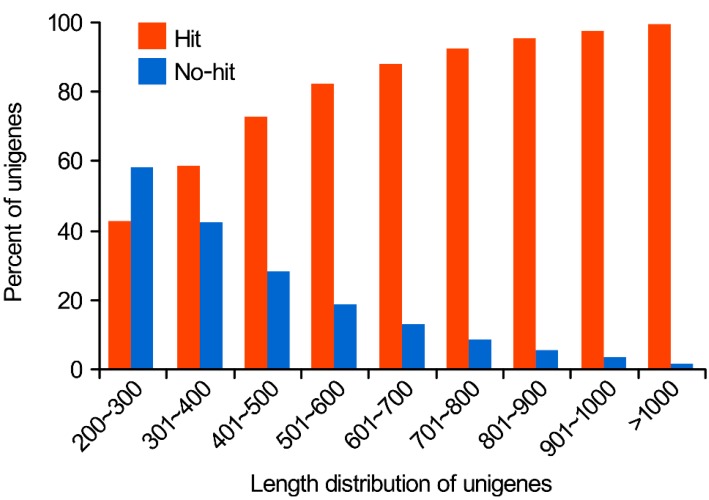
Comparison of unigene length between hit and no-hit unigenes in Nr database.

To retrieve proteins with the highest sequence similarity, distinct unigenes were aligned to Nr and Swiss-prot databases using Blastx algorithm (*E*-value < 1 × 10^−5^). Out of 71,095 all-unigenes from Pg-Con and Pg-MeJA, 54,920 (77.25%) were found to have significant similarity to 12,416 protein accessions in Nr database, whereas 35,448 (49.92%) with significant identities to Swiss-prot database were matched with 22,198 protein accessions. It was reported that longer contigs were more likely to show Blast matches in the protein databases [27]. Our results also showed that 94% of unigenes over 500 bp in length had Blast matches in the Nr database, while only 42% of unigenes shorter than 300 bp did (Figure 1).

**Figure 2:**
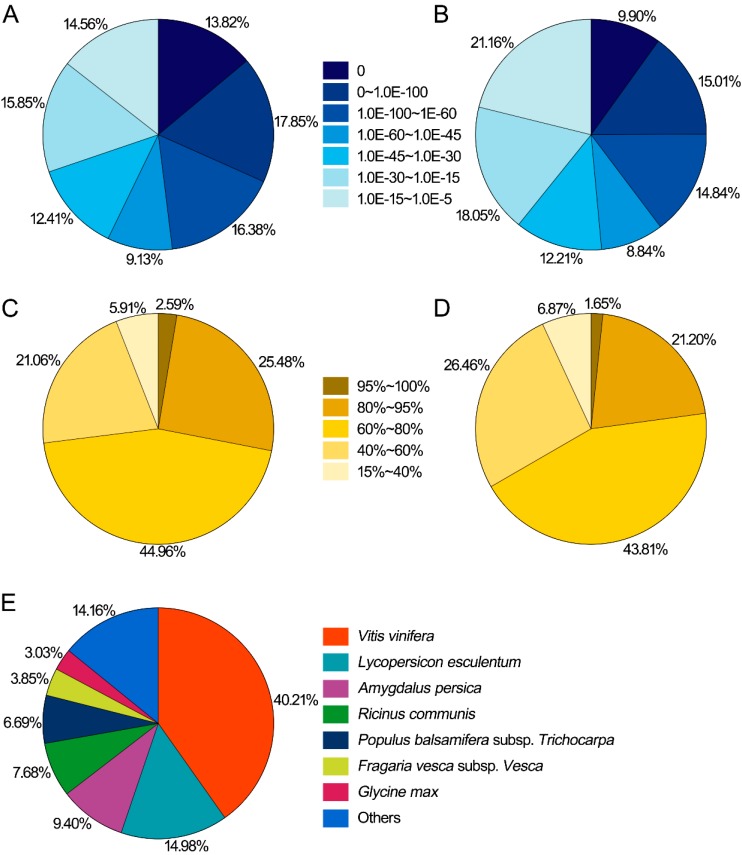
Searching characterization of the assembled unigenes against Nr and Swiss-prot databases. (**A**) *E*-value distribution of Blast hits in Nr database; (**B**) *E*-value distribution of Blast hits in Swiss-prot database; (**C**) Distribution of assembled unigene similarities based on the top Blast hits in Nr database; (**D**) Distribution of assembled unigene similarities based on the top Blast hits in Swiss-prot database; and (**E**) Homology distribution among other plant species based on the top Blast hits in Nr database.

In the Nr database, most of the matched sequences (85.44%) had *E*-values between 0 and 1 × 10^−15^ and the rest 14.56% had *E*-values from 1 × 10^−15^ to 1 × 10^−5^ (Figure 2A). Likewise, the *E*-value distribution in the Swiss-prot database revealed that the majority of matched sequences (78.84%) had *E*-values from 0 to 1 × 10^−15^ and the remaining 21.16% had the *E*-values between 1 × 10^−15^ and 1 × 10^−5^ (Figure 2B). The translated amino acid sequences of unigenes showed a high similarity to the sequences from the Nr database. Nearly 94.09% of the Blastx hits were in a similarity range between 40% and 100% and only 5.91% of hits had similarity values less than 40% (Figure 2C). The similarity distribution in the Swiss-prot was also more similar to that of the Nr database. Similarities between 40% and 100% were found for 93.13% of query sequences against Swiss-prot, whereas only 6.87% had lower homologies with <40% identity (Figure 2D). Homologies among different species are illustrated in Figure 2E, where out of the matched 54,920 unigenes in Nr, 22,083 (40.21%) were matched to *V. vinifera* followed by *Lycopersicon esculentum* (8226; 14.98%) and *Amygdalus persica* (5161; 9.40%). Thus *P. ginseng* was genetically more related to *V. vinifera*.

#### 2.1.3. GO (Gene Ontology) Functional Classification

**Figure 3:**
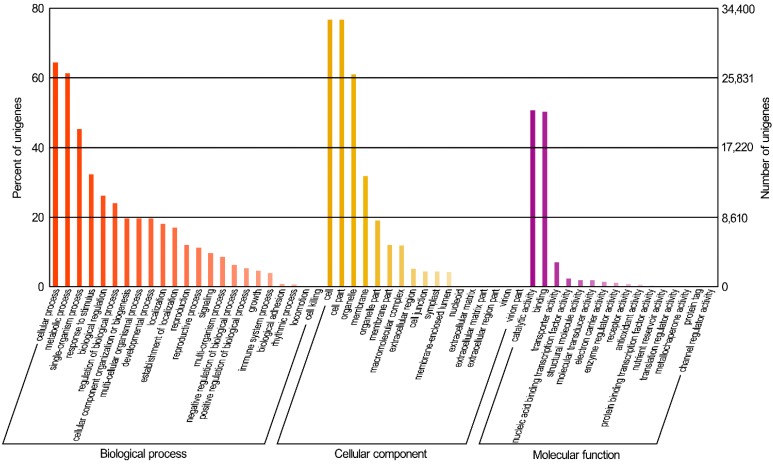
Histogram presentation of gene ontology (GO) functional classification. The results are summarized in three GO ontologies: biological process, cellular component and molecular function. The right y-axis indicates the number of unigenes in a category and the left y-axis indicates the percentage in the same category.

GO assignments were used to classify the predicted functions of *P. ginseng* adventitious root genes. In our work, the Blast2GO program [28] was used first to obtain GO annotation of assembled unigenes annotated by Nr and then the WEGO software [29] was utilized to perform GO functional classification of the unigenes. In total, 43,051 unigenes with Blast matches to known proteins were assigned to the three GO ontologies that were further sub-divided into 46 categories (Table S1). As shown in Figure 3, assignments to the cellular component ranked the highest (34,946; 81.17%), followed by biological process (33,708; 78%) and molecular function (32,970; 76.58%). Under biological process, cellular process (64.44%) and metabolic process (61.34%) were prominently represented indicating that some important cellular processes and metabolic activities occurred in *P. ginseng* adventitious roots. Under the category of cellular components, cell (76.67%), cell part (76.67%) and organelle (61.10%) represented the majorities of the category. Catalytic activity (50.70%) and binding (50.34%) were the most abundant among various molecular functions. However, we found only a small number of genes in the categories of cell killing (1 unigene), channel regulator activity (1 unigene), protein tag (3 unigenes) and metallochaperone activity (10 unigenes). 

#### 2.1.4. COG (Clusters of Orthologous Groups) Functional Classification

**Figure 4:**
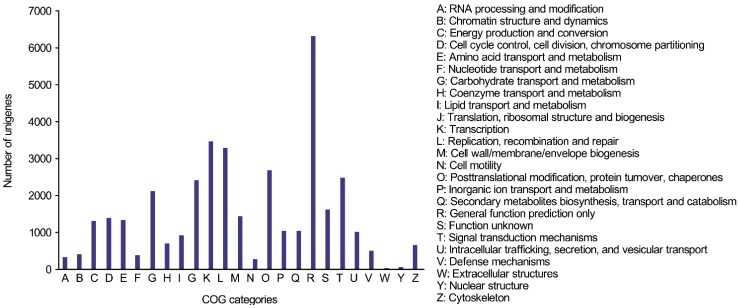
Histogram presentation of clusters of orthologous groups (COG) functional classification.

In our study, all annotated uningenes were aligned to the COG database to further assess the completeness of transcriptome library and the effectiveness of annotation process. Out of the 71,095 all-unigenes, 19,986 were assigned to 25 COG categories (Figure 4, Table S2), where “general function prediction only” category represented the largest group (6308; 31.56%) followed by “transcription” (3459; 17.31%), “replication, recombination and repair” (3275; 16.39%) and “posttranslational modification, protein turnover, chaperones” (2672; 13.37%). The following categories: “extracellular structures” (22 unigenes) and “nuclear structure” (43 unigenes) represented the smallest groups. In addition, 1029 unigenes were assigned to “secondary metabolites biosynthesis, transport and catabolism” suggesting that these important processes might take place in *P. ginseng* adventitious roots.

#### 2.1.5. KEGG (Kyoto Encyclopedia of Genes and Genomes) Pathway Annotation

**Figure 5:**
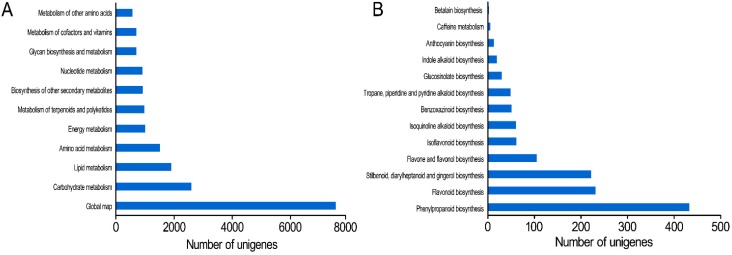
(**A**) Unigenes of 11 sub-categories under “metabolism” category; (**B**) Unigenes of 13 pathways under “other secondary metabolites biosynthesis” sub-category.

To identify the active biological pathways and for further understanding the biological functions and interactions of genes, 71,095 all-unigenes were analyzed to the KEGG pathway database. In total, 32,200 unigenes having significant matches in the database were assigned to 5 categories (level 1), 19 sub-categories (level 2) and 128 pathways (level 3) (Table S3). In level 3, “metabolic pathways” had the largest number of unigenes (7137; 22.16%) followed by “biosynthesis of secondary metabolites” (3419; 10.62%). The “metabolism” category under level 2 contained 11 sub-categories, of which the large four are “global map”, “carbohydrate metabolism”, “lipid metabolism” and “amino acid metabolism” (Figure 5A). In the “biosynthesis of other secondary metabolites” sub-category, 1280 uningenes were classified into 13 pathways and most of them were mapped to “phenylpropanoid biosynthesis”, “flavonoid biosynthesis”, “stilbenoid, diarylheptanoid and gengerol biosynthesis” and “flavone and flavonol biosynthesis” (Figure 5B). Many enzymes mapped to unigenes in KEGG pathways indicate that the active metabolic processes were underway in *P. ginseng* adventitious roots and a variety of metabolites were synthesized as well.

#### 2.1.6. Gene Expression Profiling

**Figure 6:**
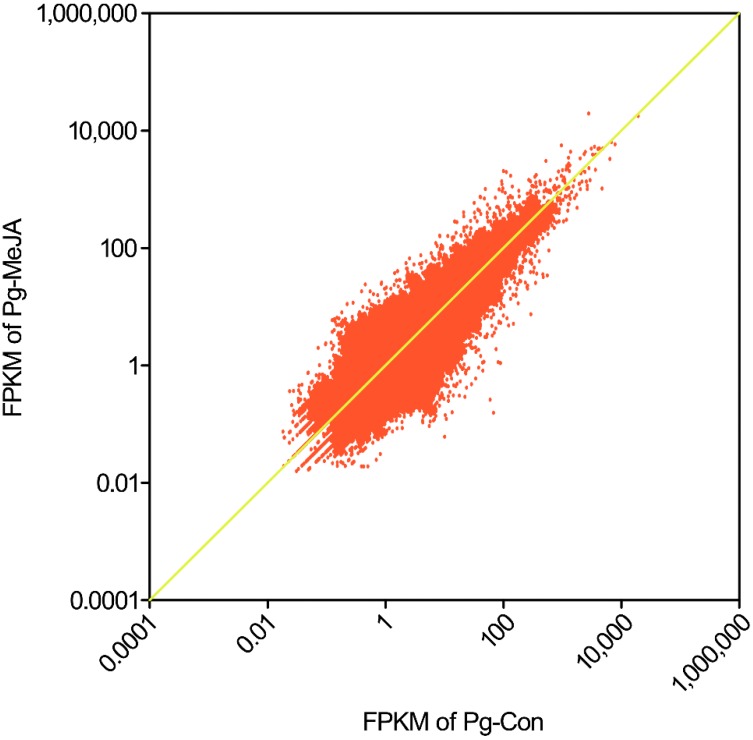
FPKM values of all-unigenes expressed in Pg-Con and Pg-MeJA. Each dot indicates one unigene. The dots above the yellow line indicate up-regulated genes and below this line are down-regulated genes. The horizontal coordinates represent the FPKM values of Pg-Con and the vertical coordinates the Pg-MeJA FPKM values. The X and Y-axis are in log-scale.

The expression levels of unigenes were represented as FPKM (fragments per kilobase per million) values based on our data produced by Illumina/Solexa platform with false discovery rate (FDR) ≤0.001. For the Pg-Con unigenes, the FPKM ranged from 0.02 to 7807.74 with an average of 14.67, while the FPKM for Pg-MeJA ranged from 0.02 to 19,547.06 with an average of 14.17. This indicates that both Pg-Con and Pg-MeJA unigenes showed a wide range of expression level implying that Illumina HiSeq™2000 could potentially identify genes with extremely low to strong expression levels in non-model plants as well. Thus, unigenes above the yellow line in Figure 6 indicate up-regulation by MeJA and below this line show down-regulation. Using FPKM values, we identified 25,999 up-regulated and 39,536 down-regulated unigenes (Table S4).

#### 2.1.7. Identification of Candidate Genes Involved in Ginsenoside Biosynthesis

**Figure 7:**
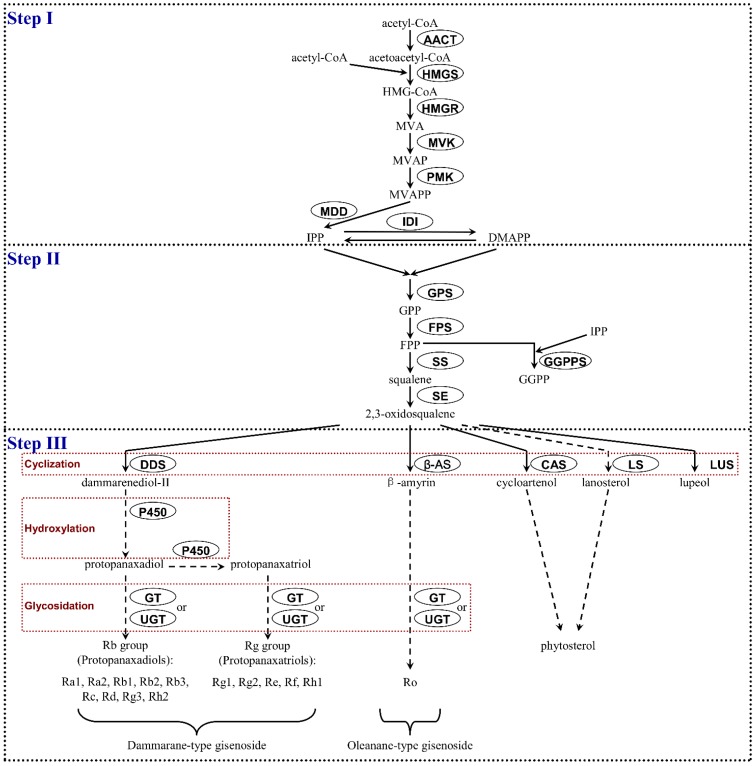
Putative ginsenoside biosynthesis pathway in *P. ginseng*. Enzymes encoded by unigenes found in this study are circled. MVA, mevalonate; MVAP, mevalonate phosphate; MVAPP, mevalonate diphosphate; IPP, isopentenyl diphosphate; DMAPP, dimethylallyl diphosphate; GPP, geranyl diphosphate; FPP, farnesyl diphosphate.

MeJA functions as an effective elicitor to boost the production of many secondary metabolites. Recent research shows that MeJA markedly promotes saikosaponin production in adventitious roots of *Bupleurum falcatum* [30] and regulates the phenolics, terpenes and alkaloids accumulation in plant cell suspension cultures [31]. Treatment of *P. ginseng* hairy roots and cultured cells by MeJA has been shown to increase ginsenoside content [22,23]. Increasing reports reveal that MeJA can up-regulate metabolite-related enzyme genes as well [32]. Supporting these premises, MeJA elicited adventitious roots of *P. ginseng* were used to screen genes related to ginsenoside biosynthesis. More recent evidence shows that both the MVA and the non-MVA (also known as MEP or DOXP) pathways are involved in ginsenoside biosynthesis. The synthesis is performed in the cytosol from acetyl-CoA via the MVA pathway and from pyruvate and glyceraldehyde-3-phosphate in the plastid via the non-MVA pathway [33]. It is noteworthy that a total of 749 unigenes (Table S5) in our Solexa/Illumina dataset encoding almost all the known enzymes were found to be involved in ginsenoside biosynthesis via the MVA pathway. However, we did not find any enzyme gene participating in the non-MVA pathway. The identified enzymes from our dataset involved from acetyl-CoA to ginsenoside biosynthesis are shown in three steps (Table 2, Figure 7).

**Table 2:** Differentially expressed unigenes encoding enzymes in the ginsenoside biosynthesis pathway.

Ginsenoside Biosynthesis Steps	Name of Enzymes	Number of Unigenes	Up-Regulated	Down-Regulated	Not DEGs
Step I	AACT	3	1	2	-
	HMGS	9	2	7	-
	HMGR	15	4	11	-
	MVK	20	14	6	-
	PMK	16	10	6	-
	MDD	2	1	1	-
	IDI	2	1	1	-
Step II	GPS	24	8	16	-
	FPS	1	1	0	-
	GGPPS	14	6	8	-
	SS	10	10	0	-
	SE	11	7	4	-
Step III	DDS	1	1	0	-
	β-AS	9	2	7	-
	CAS	17	1	16	-
	LS	2	0	2	-
	P450	335	161	168	6
	GT	142	48	92	2
	UGT	116	71	45	-

AACT, acetyl-CoA acetyltransferase; HMGS, HMG-CoA synthase; HMGR, HMG-CoA reductase; MVK, mevalonate kinase; PMK, phosphomevalonate kinase; MDD, mevalonate-5-diphosphate decarboxylase; IDI, isopentenyl diphosphate isomerase; GPS, geranyl diphosphate synthase; FPS, farnesyl diphosphate synthase; GGPPS, geranylgeranyl diphosphate synthase; SS, squalene synthase; SE, squalene epoxidase; DDS, dammarenediol synthase; β-AS, beta-amyrin synthase; CAS, cycloartenol synthase; LS, lanosterol synthase; P450, cytochrome P450; GT, glycosyltransferase; UGT, UDP-glycosyltransferase; DEGs, differentially expressed genes.

**Step I**: Genes encoding all the ginsenoside biosynthetic enzymes involved in this step were found in our study including acetyl-CoA acetyltransferase (AACT), HMG-CoA synthase (HMGS), HMG-CoA reductase (HMGR), mevalonate kinase (MVK), phosphomevalonate kinase (PMK), mevalonate diphosphate decarboxylase (MDD) and isopentenyl diphosphate isomerase (IDI). Here, MVK catalyzing the production of mevalonate phosphate ranked the highest (20 unigenes) and most of them (14 unigenes) were found to be up-regulated. However, no *MVK* gene was previously detected in untreated 11-year-old *P. ginseng* [34] and 4-year-old American ginseng root [35], with the exception of only 3 candidate genes in 4-year-old untreated root of *P. ginseng* [15]. HMGS involved in this step was not previously discovered from 4-year-old of *P. notoginseng* [16] and *P. quinquefolius* [35] untreated root, while we obtained 9 candidates from our work. The difference of the above results may be due to the MeJA induction or the type of organs, or species. IDI catalyzes the reversible conversion of isopentenyl diphosphate (IPP) and its isomer dimethylallyl diphosphate (DMAPP) that are the universal precursors for isoprenoids biosynthesis including ginsenosides in all organisms [36]. So far, one *IDI* candidate from the 454-EST dataset of 4-year-old untreated *P. ginseng* root was identified [15]. However, no *IDI* was discovered from MeJA-treated hairy roots of 4-year-old *P. ginseng* using traditional Sanger EST analyses [10]. But two unigenes encoding IDI were found from our dataset that may indicate the high coverage of Illumina/Solexa sequencing platform.

**Figure 8:**
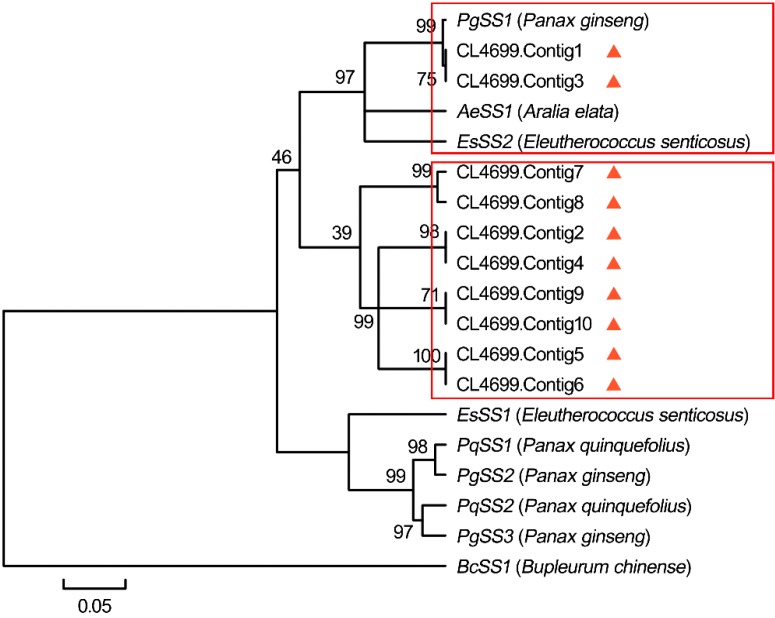
Phylogenetic analysis of 10 identified *SS* unigenes from our dataset and other plant *SS*s. Phylogenetic tree was generated using the neighbor-joining (NJ) method in MEGA4. ▲ indicates the unigenes from our current study. The box in red outline indicates one group of genes that are more similar with each other.

**Step II**: We identified several candidate genes encoding geranyl diphosphate synthase (GPS), farnesyl diphosphate synthase (FPS), squalene synthase (SS) and squalene epoxidase (SE) involved from IPP to 2,3-oxidosqualene. In a previous study, two unigenes for GPS in 4-year-old *P. ginseng* root were detected [15], while no *GPS* gene for this enzyme was found in 11-year-old *P. ginseng* [34]. Here, 24 *GPS* unigenes were discovered including eight up-regulated and 16 down-regulated candidates. In a study, the expression of the *FPS* was shown to be up-regulated by MeJA in *P. ginseng* hairy roots [37]. We found one *FPS* unigene from our dataset that was also up-regulated. FPP is converted to squalene under the action of SS. This is the first enzymatic reaction from the central isoprenoid pathway towards sterol and triterpene biosynthesis [11] and may be a potential point for their biosynthesis regulation. Recent studies showed that only one unigene encoding SS was found in both 4- and 11-year old untreated *P. ginseng* root [15,34]. In another case, two up-regulated *SS* candidates were discovered from MeJA-treated hairy roots based on Sanger EST analyses [10]. It is noteworthy that 10 *SS* candidates (Table S6) encoding SS enzymes for squalene synthesis were found in this study and all of them were up-regulated, indicating that squalene production was very active in our 4-year MeJA-treated *P. ginseng* adventitious roots. Phylogenetic analysis revealed that some *SS* genes closely formed one branch in the tree, even though they were derived from different plant species (Figure 8). In particular, two contigs (CL4699.contig1 and CL4699.contig3) out of 10 belonging to cluster CL4699 showed close similarity to the *P. ginseng* candidate *SS1* up-regulated by MeJA [37] indicating that these contigs were likely to be involved in ginsenoside biosynthesis. SE converts squalene to 2,3-oxidosqualene from which phytosterol and triterpene in plants are synthesized. The number of unigenes for SE was larger in our work than that of previously identified by the 454 pyrosequencing and Sanger EST analyses in untreated and MeJA-treated *P. ginseng* root respectively [15,34]. These results may represent the immense capacity of both MeJA and Illumina/Solexa sequencing technology. 

**Step III**: In *P. ginseng*, dammarenediol synthase (DDS), β-amyrin synthase (β-AS), cycloartenol synthase (CAS), lanosterol synthase (LS) and lupeol synthase (LUS) belonging to oxidosqualene cyclase (OSC) family is situated at the branching point of ginsenoside and phytosterol biosynthesis. Their biosynthesis is achieved by three reaction steps namely cyclization, hydroxylation and glycosidation [38]. In cyclization, 2,3-oxidosqualene is converted to cycloartenol by CAS and lanosterol by LS. 2,3-oxidosqualene is also converted to β-amyrin under the action of β-AS and subsequently oleanane-type ginsenoside (Ro) is synthesized. However, the absence of β-AS and CAS both in 4- and 11-year old *P. ginseng* roots [15,34] may indicate that oleanane-type ginsenoside (Ro) and phytosterol were not actively biosynthesized in their roots. Another branch of this step is the DDS enzyme catalyzing the production of dammarenediol-II from 2,3-oxidosqualene. The genes encoding this enzyme were shown to be up-regulated by MeJA [37,39]. DDS was shown to be absent in 11-year-old *P. ginseng* root [34] indicating that dammarane-type ginsenosides biosynthesis was not active in its roots. Moreover, many genes were discovered from transcriptome analysis of 4-year-old American ginseng roots. However, no gene responsible for the cyclization of oxidosqualene was found [35]. Interestingly, suppression of CAS caused enhanced ginsenoside content in *P. ginseng* hairy roots. This phenomenon was related to lower CAS and higher DDS activity, presumably due to increased availability of 2,3-oxidosqualene for DDS to produce dammarenediol-II and subsequently dammarane-type ginsenosides [40]. We have identified one candidate encoding DDS induced by MeJA induction. However, out of a total 17 *CAS* and 2 *LS* unigenes found in our dataset, 16 candidates encoding CAS and all of the *LS* unigenes were down-regulated indicating that lower CAS and LS activity may supply more 2,3-oxidosqualene for DDS and β-AS leading to the increased final ginsenoside levels and reduced total phytosterol contents. However, we did not find any LUS functioning for lupeol synthesis indicating that its absence may increase the final ginsenoside levels as well.

In hydroxylation, two reaction steps are catalyzed by two cytochrome P450 (P450) enzymes: the conversion from dammarenediol-II to protopanaxadiol by one P450 and the conversion from protopanaxadiol to protopanaxatriol by another P450 [41,42]. One hundred and thirty-three *P450*s and 25 *P450*s candidates were identified previously in a cDNA library generated from 4-year-old MeJA-induced and 11-year-old untreated hairy roots of *P. ginseng* respectively [10,34]. In another case, among 27 *P450*s, six were up-regulated and eight were down-regulated in response to MeJA [17]. By comparison, we found a total of 335 *P450*s unigenes in our study; of those 161 were up-regulated and 168 down-regulated and six candidates did not show any differential expression level. This large number of *P450* candidates gives a prospective gene resource in the detection of special P450s related to ginsenoside biosynthesis in *P. ginseng*.

Glycosidation occurs in the last reaction step where different ginsenosides are synthesized by adding one or several monosaccharides to triterpene aglycones by UDP-glycosyltransferase (UGT) [43,44]. 235 *UGT*s from 11-year-old *P. ginseng* root transcriptome have previously been identified [34]. Among 27 *UGT*s discovered in *P. quinquefolius*, 11 *UGT*s were up-regulated and three were down-regulated by MeJA [17]. In this study, a total of 116 unigenes encoding UGTs were identified, of which 71 candidates were up-regulated and 45 were down-regulated in response to MeJA. These observations show that MeJA as a signal transducer leads to the regulation of the enzyme genes involved in ginsenoside biosynthesis.

#### 2.1.8. Identification of Candidate Genes Involved in Ginsenoside Transport

**Figure 9:**
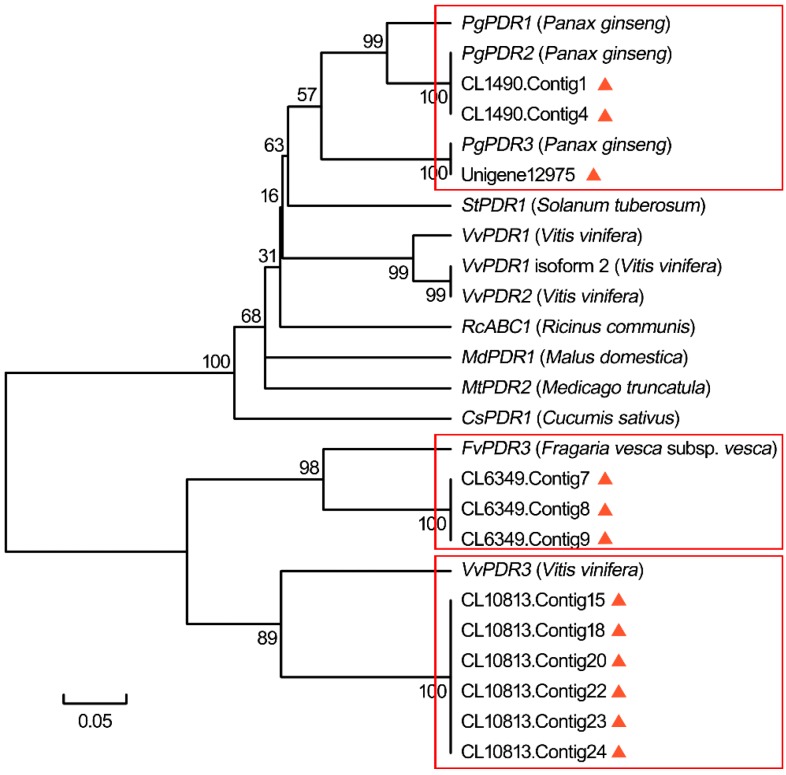
Phylogenetic analysis of 12 potential *PDR* unigenes of our dataset and other plant *PDRs*. Among two kinds of unigenes, some having 70% similarity among them, are under one cluster (CL) initiated with “CL” along with the same number such as “CL1490”, followed by different number of contigs, whereas others start directly with “Unigene” such as “Unigene12975”. ▲ indicates the unigenes from our current study. The box in red outline indicates one group of genes that are more similar with each other.

Interestingly, MeJA may enhance transporter synthesis to assist metabolite transport in a temporal and spatial manner in plant organs [45]. Moreover, as compared to ginsenoside biosynthesis, little is known about ginsenoside transport and regulation in cells. Therefore, we have also focused on detecting a number of genes related to the transport of secondary metabolites through plant PDR transporters belonging to the ABCG subfamily of ATP-binding cassette (ABC) transporters. Among several full length ABC subfamilies, the best-characterized three subfamilies are PDR, multidrug resistance (MDR) and multidrug resistance-associated protein (MRP) involved in the transport of various molecules across membranes [46,47]. In the present study, many ABC members such as *MDR*, *MRP* and *PDR* genes along with their expression were screened from our transcriptome dataset. Among the unigene hits, a total of 595 unigenes for ABC (from ABCA to ABCG), 229 for MDR, 76 for MRP and 125 for PDR were found (Tables S7–S10). Further evidence demonstrates that PDR transporters take part in exporting a wide range of substrates across membranes in different environmental conditions [46]. In our previous study, three *PgPDR* genes (*PgPDR1*, *PgPDR2* and *PgPDR3)* were isolated from *P. ginseng*, where *PgPDR3* was induced by MeJA and its expression was highly related to ginsenoside accumulation. Our findings show that *PgPDR3* participates in the export of ginsenosides from the cells [19,25]. Of the identified 125 *PDR* unigenes in the current study, 70 were up-regulated by MeJA, such as the three *PgPDRs* (mentioned above). Homology and similarity analysis shown in Figure 9 and Table S6 provided 12 promising *PDR* candidates out of 125 *PDR* unigenes. Of those, in particular three unigenes, “CL1490.Contig1”, “CL1490.Contig4” and “Unigene12975” showed a much higher degree of similarity to the three *PgPDR* genes, indicating that they were most likely to be involved in the export of ginsenosides. Likewise, we found three contigs of “CL6349” and six of “CL10813” from our dataset closely related to *FvPDR3* of *Fragaria vesca* subsp. *Vesca* and *VvPDR3* of *V. vinifera* respectively. With respect to these points of view, our MeJA-treated sequencing results provided a multitude potential genes associated with the transport of a wide range of substrates including ginsenosides and other secondary metabolites.

### 2.2. Discussion

Transcriptome sequencing is one of the most effective tools for gene identification. Understanding the transcriptome analysis is essential to know which genes are expressed at various developmental stages or physiological conditions of a cell. Plant genomics, especially for non-model species, is always hard due to its large and complex genome, high levels of ploidy and large proportions of repeat sequences. Instead of the whole genome sequencing, transcriptome sequencing is a better alternative for rapid and efficient access to genetic information [48]. High-throughput Illumina/Solexa sequencing technology has recently been an efficient, fast, inexpensive and reliable tool for transcriptome characterization and gene discovery in non-model organisms as well. Previously, a number of ESTs were generated from leaves, seeds, rhizomes and roots of *P. ginseng* by the Sanger sequencing technology [11,12,49,50]. However, deep EST sequencing using this traditional method is highly expensive, labor intensive and time-consuming. Therefore, with the development of large scale genomics, the Sanger method could not meet the needs of the development. However, over the past few years, the NGS technologies have overcome the current limitations of the Sanger method and at the same time, the NGS platform has become the prime approach for high-throughput gene discovery on a genome-wide scale both for model and non-model organisms. These technologies can produce a large number of quality reads with high sampling rates of cDNA libraries providing a complete view of transcriptomes. Thus, plant genomics in recent years has developed rapidly with the application of various NGS technologies with respect to costs, efficiency and speed compared with that of the traditional Sanger method. Hence, over the past several years, these technologies have made a revolution in genomics and provided a rapid and economic way to sequence extremely large amounts of genetic material [51,52]. Among NGS technologies, the Illumina/Solexa platform has been one of the most useful and widely used technologies due to its fast, inexpensive, accurate, efficient and reliable transcriptome characterization, gene discovery and various functional studies in many organisms [53]. Using this technology, comprehensive sequencing resources have been successfully constructed from non-model plants including *P. ginseng* [14], *Lentinula edodes* [54] and *Auricularia polytricha* [55]. However, transcriptome assembly was highly challenging including mis-assembled contigs [56]. Recently, “Trinity”, [26] a novel assembly method has been introduced most notably through an expand capability for transcriptome *de novo* assembly using relatively short reads. The results from this study suggested that short reads from Illumina/Solexa sequencing can effectively be assembled through “Trinity” giving the advantage of a high assurance and reliable assembly of putative unigenes, including potential and valuable splice variant information. Therefore, *de novo* sequencing and assembly of transcriptomes or genomes has effectively been utilized for model [57,58] and non-model organisms [59,60,61].

Despite the commercial and medicinal values of *P. ginseng*, NGS-based MeJA-elicited transcriptome information on this species in public databases was scarce prior to our study. In our work, Illumina/Solexa sequencing technology was applied to perform this work. Compared to the Sanger method, this technology provides a large amount of genetic resources in a fast, efficient and economic way. Through this program, we obtained about 55 millions raw reads separately from Pg-Con and Pg-MeJA and subsequently 53 millions clean reads from each sample. Finally, all of these clean reads assembled into 71,095 all-unigenes were annotated with five above mentioned main databases (Table S11). Furthermore, the Trinity assembly algorithm designed specifically for assembling of putative transcripts has also given us biologically significant results of high confidence. More recent works obtained similar advantages to the use of the Illumina HiSeq™2000 platform in the assembly of a transcriptome for untreated *P. ginseng* adventitious roots and *Lentinula edodes* C_91-3_ respectively [14,54].

More importantly, stimulation of ginsenoside synthesis by MeJA is performed by the up-regulation of genes including *SS*, *SE*, and *DDS* involved in ginsenoside biosynthesis [37,62]. Although many candidate genes related to ginsenoside biosynthesis have previously been identified both in untreated and MeJA treated *P. ginseng*, several genes encoding ginsenoside biosynthetic enzymes such as *MVK*, *GPS*, *β-AS* and *DDS* were not found [10,34]. For example, DDS thought to be a rate-limiting and the first enzyme in the biosynthetic pathway of dammarane-type saponins was absent in the 454 EST dataset of untreated *P. ginseng* roots [34]. Some recent works have discovered only one unigene from 4-year-old untreated *P. ginseng* [15] and three unigenes from American ginseng roots [17] based on the 454 pyrosequencing method. In another case, only two up-regulated *SS* candidates were found from the same year-old MeJA-treated *P. ginseng* hairy roots via the traditional Sanger EST analyses [10]. However, we have obtained 10 up-regulated *SS* unigenes from our dataset. Moreover, nearly all types of major ginsenoside biosynthetic enzyme genes were discovered in the transcriptome of our MeJA-treated *P. ginseng* adventitious roots (mentioned above). Based on comparison, the findings indicate that the information of this study is relatively comprehensive and highlights the immense capacity of both MeJA and Illumina/Solexa technology. On the other hand, only a few ABC transporters have been identified in medicinal plants related to ginsenosides and transport of other secondary metabolites. Moreover, several MeJA-induced plant PDRs have been discovered associated with defense reactions against pests, pathogens and herbivores by transporting antimicrobial natural secondary metabolites in cells [63,64,65]. Based on the knowledge that secondary metabolites can be induced by MeJA, the identified 12 potential candidate *PDR* unigenes from our study through phylogenetic analysis will provide more important functions related to the transport of various substrates including secondary metabolites and their transport in cells. Moreover, the annotated unigenes and associated resources from our work provide valuable information for *P. ginseng* adventitious roots to investigate specific processes and allow the identification of novel genes related to ginsenosides and other secondary metabolites biosynthesis, their accumulation, as well as transport, that are responsive to MeJA treatment.

## 3. Experimental Section

### 3.1. Preparation of Plant Materials

Chinese Jilin ginseng cv. Damaya cultivated mainly in the shade is a cold resistant and fast growing variety of *P. ginseng* with a high content of ginsenosides. Commercially cultivated 4-year-old *P. ginseng* (Damaya) was collected on the 28 July 2013 from the forest of the Changbai mountain of Fusong county, Baishan, China. The fresh roots were sterilized as described earlier [23], cut into small pieces and then cultured in solid MS media supplemented with 1.0 mg·L^−1^ 2,4-dichlorophenoxy acetic acid (2,4-D) and 0.1 mg·L^−1^ kinetin (KT) and kept 28 days at 25 °C in dark for callus induction. Afterwards, the callus was transferred to new solid MS media supplemented with 5.0 mg·L^−1^ indole-3-butyric acid (IBA) and kept 28 days at 25 °C in the dark for adventitious roots induction. Newly established adventitious roots were moved to liquid MS media supplemented with 5.0 mg·L^−1^ IBA and maintained on a rotary shaker at 160 rpm and 25 °C in the dark and then sub-cultured once every 2 weeks. MeJA (200 µM, desired concentration) was added to the 2 weeks cultured adventitious roots for 24 h (Pg-MeJA). Equal volumes of solvent (ethanol) was dissolved in the experimental controls and kept in the same condition (Pg-Con).

### 3.2. RNA Extraction and cDNA Library Construction for Sequencing

The total RNA from two samples (Pg-Con and Pg-MeJA) was extracted with E.Z.N.A.^®^ Plant RNA Kit (Omega Bio-Tek, Doraville, GA, USA) following the manufacturer’s procedure and then treated with DNaseI to remove DNA residues. RNA (31.26 µg) at a concentration of 665 ng·μL^−1^ from Pg-Con and 30.40 µg at a concentration of 351 ng·μL^−1^ from Pg-MeJA were used for cDNA library preparation. For Illumina/Solexa sequencing, mRNA with poly(A) tail was isolated from total RNA using oligo(dT) magnetic beads. Poly(A) RNA was then purified and fragmented into small pieces by using divalent cations at elevated temperature. Then double-stranded cDNA was synthesized from cleaved mRNA fragments. Short cDNA fragments were purified and resolved with Elution Buffer (10 mM Tris-HCl, pH 8.5) for end reparation and poly(A) addition. After that, the short cDNA fragments were attached with sequencing adapters. For PCR amplification, appropriate fragments were selected as templates based on the agarose gel electrophoresis result. Finally, the cDNA library products were sequenced using Illumina HiSeq™2000 at BGI (http://www.genomics.cn/en/index), Shenzhen, China.

### 3.3. Data Filtering and Sequence Assembly

Prior to assembly, raw reads including adaptors, and unknown or low quality bases were filtered to obtain high-quality transcriptome sequence data. *De novo* assembly of the short clean reads was carried out by assembling program “Trinity” (http://trinityrnaseq.sourceforge.net) [26]. The resulting sequences from “Trinity” are called unigenes. If multiple samples from the same species were sequenced, unigenes from each sample’s assembly can be taken into further process of sequence splicing and redundancy, removing with sequence clustering software to get non-redundant unigenes called all-unigene.

### 3.4. Protein Coding Sequence (CDS) Prediction

Blastx alignment (*E*-value < 1 × 10^−5^) between public databases like Nr (http://www.ncbi.nlm.nih.gov), Swiss-prot (http://www.expasy.ch/sprot), KEGG (http://www.genome.jp/kegg) and COG (http://www.ncbi.nlm.nih.gov/cog) were performed and the best aligning results were used to decide sequence direction (5'–3') of unigenes. If the searching results obtained from different databases conflicted with each other, a priority order of Nr, Swiss-prot, KEGG and COG would have been followed while deciding sequence direction of unigenes. When some unigene happened to be unaligned to none of the above mentioned databases, “ESTScan” (www.ch.embnet.org/software/ESTScan.html) [66] was used to predict its coding regions and to decide the sequence direction.

### 3.5. Unigene Annotation and Classification

The annotation of unigenes was carried out through a variety of bioinformatics procedures using Blastx (*E*-value < 1 × 10^−5^). With Nr annotation, the Blast2GO program [28] was applied to obtain GO annotation of unigenes according to molecular function, biological processes and cellular component. After obtaining GO annotation for every unigene, WEGO software [29] was used to perform GO functional classification for all the annotated unigenes and to understand the distribution of species’ gene functions at the macro level. The COG database was applied to classify orthologous gene products. We therefore aligned unigenes to the COG database to classify and predict the possible functions of the unigenes. The KEGG database helps to analyze the gene product during the metabolism process and gene function in the cellular processes. So, we used the KEGG annotation to assign the pathway annotations of the unigenes.

## 4. Conclusions

Recently, with the application of NGS, genomic information of medicinal plants has developed rapidly. Such invaluable information is useful for the development of herbal medicines. However, MeJA-elicited insufficient transcriptomic and genomic data on *P. ginseng* in the public databases has limited our understanding of the molecular mechanism underlying gene discovery and gene profiling. Biosynthesis of many types of secondary metabolites including ginsenosides and their transport and accumulation are induced by MeJA [67]. Thus, the study was conducted on the MeJA-treated highly valued *P. ginseng* roots containing ginsenosides. Seventy-one thousand and ninety-five unigenes obtained from Illumina/Solxa sequencing demonstrate an imperative transcriptomic level of information for *P. ginseng* and will surely accelerate further functional genomics research for related medicinal plants as well. Additionally, from these generated sequences, a number of genes were identified encoding nearly all the known enzymes related to ginsenoside biosynthesis. More importantly, a handful of candidate *PDR* genes were discovered playing important roles in the transport of secondary metabolites including ginsenosides in medicinal plants. Furthermore, our results also represent an imperative capacity of Illumina/Solexa sequencing as a fast, reliable, less experimental complexity and cost-effective approach for transcriptome characterization and gene discovery in non-model organisms, especially those with complex and large genomes such as *P. ginseng*.

## Supplementary Materials

Supplementary materials can be found at http://www.mdpi.com/1422-0067/16/02/3035/s1.

## References

[1] Wen J., Zimmer E.A. (1996). Phylogeny and biogeography of *Panax* L. (the ginseng genus, araliaceae): Inferences from ITS sequences of nuclear ribosomal DNA. Mol. Phylogenet. Evol..

[2] Yun T.K. (2001). Brief introduction of *Panax ginseng* C.A. Meyer. J. Korean Med. Sci..

[3] Yan X., Fan Y., Wei W., Wang P., Liu Q., Wei Y., Zhang L., Zhao G., Yue J., Zhou Z. (2014). Production of bioactive ginsenoside compound K in metabolically engineered yeast. Cell Res..

[4] Chu S.F., Zhang J.T. (2009). New achievements in ginseng research and its future prospects. Chin. J. Integr. Med..

[5] Zhang Z., Du G.J., Wang C.Z., Wen X.D., Calway T., Li Z., He T.C., Du W., Bissonnette M., Musch M.W. (2013). Compound K, a ginsenoside metabolite, inhibits colon cancer growth via multiple pathways including p53-p21 interactions. Int. J. Mol. Sci..

[6] Lee S., Lee M.S., Kim C.T., Kim I.H., Kim Y. (2012). Ginsenoside Rg3 reduces lipid accumulation with AMP-activated protein kinase (AMPK) activation in HepG2 cells. Int. J. Mol. Sci..

[7] Kim S.J., Murthy H.N., Hahn E.J., Lee H.L., Paek K.Y. (2007). Parameters affecting the extraction of ginsenosides from the adventitious roots of ginseng (*Panax ginseng* C.A. Meyer). Sep. Purif. Technol..

[8] Chen C.F., Chiou W.F., Zhang J.T. (2008). Comparison of the pharmacological effects of *Panax ginseng* and *Panax quinquefolium*. Acta Pharmacol. Sin..

[9] Chen S.L., Sun Y.Z., Xu J., Luo H.M., Sun C., He L., Cheng X.L., Zhang B.L., Xiao P.G. (2010). Strategies of the study on herb genome program. Acta Pharmacol. Sin..

[10] Choi D.W., Jung J., Ha Y.I., Park H.W., In D.S., Chung H.J., Liu J.R. (2005). Analysis of transcripts in methyl jasmonate-treated ginseng hairy roots to identify genes involved in the biosynthesis of ginsenosides and other secondary metabolites. Plant Cell Rep..

[11] Sathiyamoorthy S., In J.G., Gayathri S., Kim Y.J., Yang D.C. (2010). Generation and gene ontology based analysis of expressed sequence tags (EST) from a *Panax ginseng* C.A. Meyer roots. Mol. Biol. Rep..

[12] Sathiyamoorthy S., In J.G., Gayathri S., Kim Y.J., Yang D. (2010). Gene ontology study of methyl jasmonate-treated and non-treated hairy roots of *Panax ginseng* to identify genes involved in secondary metabolic pathway. Genetika.

[13] Schuster S.C. (2008). Next-generation sequencing transforms today’s biology. Nat. Methods.

[14] Jayakodi M., Lee S.C., Park H.S., Jang W., Lee Y.S., Choi B.S., Nah G.J., Kim D.S., Natesan S., Sun C. (2014). Transcriptome profiling and comparative analysis of *Panax ginseng* adventitious roots. J. Ginseng Res..

[15] Li C., Zhu Y., Guo X., Sun C., Luo H., Song J., Li Y., Wang L., Qian J., Chen S. (2013). Transcriptome analysis reveals ginsenosides biosynthetic genes, microRNAs and simple sequence repeats in *Panax ginseng* C.A. Meyer. BMC Genomics.

[16] Luo H., Sun C., Sun Y., Wu Q., Li Y., Song J., Niu Y., Cheng X., Xu H., Li C. (2011). Analysis of the transcriptome of *Panax notoginseng* root uncovers putative triterpene saponin-biosynthetic genes and genetic markers. BMC Genomics.

[17] Sun C., Li Y., Wu Q., Luo H., Sun Y., Song J., Lui E.M., Chen S. (2010). *De novo* sequencing and analysis of the American ginseng root transcriptome using a GS FLX Titanium platform to discover putative genes involved in ginsenoside biosynthesis. BMC Genomics.

[18] Han J.Y., In J.G., Kwon Y.S., Choi Y.E. (2010). Regulation of ginsenoside and phytosterol biosynthesis by RNA interferences of squalene epoxidase gene in *Panax ginseng*. Phytochemistry.

[19] Zhang R., Zhu J., Cao H.Z., An Y.R., Huang J.J., Chen X.H., Mohammed N., Afrin S., Luo Z.Y. (2013). Molecular cloning and expression analysis of PDR1-like gene in ginseng subjected to salt and cold stresses or hormonal treatment. Plant Physiol. Biochem..

[20] Farmer E.E. (2007). Plant biology: Jasmonate perception machines. Nature.

[21] Wasternack C. (2007). Jasmonates: An update on biosynthesis, signal transduction and action in plant stress response, growth and development. Ann. Bot..

[22] Kim Y.S., Hahn E.J., Murthy H.N., Paek K.Y. (2004). Adventitious root growth and ginsenoside accumulation in *Panax ginseng* cultures as affected by methyl jasmonate. Biotechnol. Lett..

[23] Thanh N.T., Murthy H.N., Yu K.W., Hahn E.J., Paek K.Y. (2005). Methyl jasmonate elicitation enhanced synthesis of ginsenoside by cell suspension cultures of *Panax ginseng* in 5-l balloon type bubble bioreactors. Appl. Microbiol. Biotechnol..

[24] Crouzet J., Trombik T., Fraysse A.S., Boutry M. (2006). Organization and function of the plant pleiotropic drug resistance ABC transporter family. FEBS Lett..

[25] Zhang R., Huang J., Zhu J., Xie X., Tang Q., Chen X., Luo J., Luo Z. (2013). Isolation and characterization of a novel PDR-type ABC transporter gene *PgPDR3* from *Panax ginseng* C.A. Meyer induced by methyl jasmonate. Mol. Biol. Rep..

[26] Grabherr M.G., Haas B.J., Yassour M., Levin J.Z., Thompson D.A., Amit I., Adiconis X., Fan L., Raychowdhury R., Zeng Q. (2011). Full-length transcriptome assembly from RNA-Seq data without a reference genome. Nat. Biotechnol..

[27] Wang Z., Fang B., Chen J., Zhang X., Luo Z., Huang L., Chen X., Li Y. (2010). *De novo* assembly and characterization of root transcriptome using Illumina paired-end sequencing and development of cSSR markers in sweet potato (*Ipomoea batatas*). BMC Genomics.

[28] Conesa A., Gotz S., Garcia-Gomez J.M., Terol J., Talon M., Robles M. (2005). Blast2GO: A universal tool for annotation, visualization and analysis in functional genomics research. Bioinformatics.

[29] Ye J., Fang L., Zheng H., Zhang Y., Chen J., Zhang Z., Wang J., Li S., Li R., Bolund L. (2006). WEGO: A web tool for plotting GO annotations. Nucleic Acids Res..

[30] Aoyagi H., Kobayashi Y., Yamada K., Yokoyama M., Kusakari K., Tanaka H. (2001). Efficient production of saikosaponins in *Bupleurum falcatum* root fragments combined with signal transducers. Appl. Microbiol. Biotechnol..

[31] Gundlach H., Muller M.J., Kutchan T.M., Zenk M.H. (1992). Jasmonic acid is a signal transducer in elicitor-induced plant cell cultures. Proc. Natl. Acad. Sci. USA.

[32] Zhao C.L., Cui X.M., Chen Y.P., Liang Q. (2010). Key enzymes of triterpenoid saponin biosynthesis and the induction of their activities and gene expressions in plants. Nat. Prod. Commun..

[33] Zhao S., Wang L., Liu L., Liang Y., Sun Y., Wu J. (2014). Both the mevalonate and the non-mevalonate pathways are involved in ginsenoside biosynthesis. Plant Cell Rep..

[34] Chen S., Luo H., Li Y., Sun Y., Wu Q., Niu Y., Song J., Lv A., Zhu Y., Sun C. (2011). 454 EST analysis detects genes putatively involved in ginsenoside biosynthesis in *Panax ginseng*. Plant Cell Rep..

[35] Wu Q., Song J., Sun Y., Suo F., Li C., Luo H., Liu Y., Li Y., Zhang X., Yao H. (2010). Transcript profiles of *Panax quinquefolius* from flower, leaf and root bring new insights into genes related to ginsenosides biosynthesis and transcriptional regulation. Physiol. Plant.

[36] Liang Y., Zhao S. (2008). Progress in understanding of ginsenoside biosynthesis. Plant Biol..

[37] Kim O.T., Bang K.H., Kim Y.C., Hyun D.Y., Kim M.Y., Cha S.W. (2009). Upregulation of ginsenoside and gene expression related to triterpene biosynthesis in ginseng hairy root cultures elicited by methyl jasmonate. Plant Cell Tissue Organ Cult..

[38] Xu R., Fazio G.C., Matsuda S.P. (2004). On the origins of triterpenoid skeletal diversity. Phytochemistry.

[39] Han J.Y., Kwon Y.S., Yang D.C., Jung Y.R., Choi Y.E. (2006). Expression and RNA interference-induced silencing of the dammarenediol synthase gene in Panax ginseng. Plant Cell Physiol..

[40] Liang Y., Zhao S., Zhang X. (2009). Antisense suppression of cycloartenol synthase results in elevated ginsenoside levels in *Panax ginseng* hairy roots. Plant Mol. Biol. Rep..

[41] Nelson D.R., Ming R., Alam M., Schuler M.A. (2008). Comparison of cytochrome P450 genes from six plant genomes. Trop. Plant Biol..

[42] Coon M.J. (2005). Cytochrome P450: Nature’s most versatile biological catalyst. Annu. Rev. Pharmacol. Toxicol..

[43] Gachon C.M., Langlois-Meurinne M., Saindrenan P. (2005). Plant secondary metabolism glycosyltransferases: The emerging functional analysis. Trends Plant Sci..

[44] Lairson L.L., Henrissat B., Davies G.J., Withers S.G. (2008). Glycosyltransferases: Structures, functions, and mechanisms. Annu. Rev. Biochem..

[45] Yazaki K. (2006). ABC transporters involved in the transport of plant secondary metabolites. FEBS Lett..

[46] Nuruzzaman M., Zhang R., Cao H.Z., Luo Z.Y. (2014). Plant pleiotropic drug resistance transporters: Transport mechanism, gene expression, and function. J. Integr. Plant Biol..

[47] Kretzschmar T., Kohlen W., Sasse J., Borghi L., Schlegel M., Bachelier J.B., Reinhardt D., Bours R., Bouwmeester H.J., Martinoia E. (2012). A petunia ABC protein controls strigolactone-dependent symbiotic signalling and branching. Nature.

[48] Wang L., Li P., Brutnell T.P. (2010). Exploring plant transcriptomes using ultra high-throughput sequencing. Brief. Funct. Genomics.

[49] Kim M.K., Lee B.S., In J.G., Sun H., Yoon J.H., Yang D.C. (2006). Comparative analysis of expressed sequence tags (ESTs) of ginseng leaf. Plant Cell Rep..

[50] Jung J.D., Park H.W., Hahn Y., Hur C.G., In D.S., Chung H.J., Liu J.R., Choi D.W. (2003). Discovery of genes for ginsenoside biosynthesis by analysis of ginseng expressed sequence tags. Plant Cell Rep..

[51] Morozova O., Hirst M., Marra M.A. (2009). Applications of new sequencing technologies for transcriptome analysis. Annu. Rev. Genomics Hum. Genet..

[52] Simon S.A., Zhai J., Nandety R.S., McCormick K.P., Zeng J., Mejia D., Meyers B.C. (2009). Short-read sequencing technologies for transcriptional analyses. Annu. Rev. Plant Biol..

[53] Haas B.J., Zody M.C. (2010). Advancing RNA-Seq analysis. Nat. Biotechnol..

[54] Zhong M., Liu B., Wang X., Liu L., Lun Y., Li X., Ning A., Cao J., Huang M. (2013). *De novo* characterization of *Lentinula edodes* C(91–3) transcriptome by deep Solexa sequencing. Biochem. Biophys. Res. Commun..

[55] Zhou Y., Chen L., Fan X., Bian Y. (2014). *De novo* assembly of *Auricularia polytricha* transcriptome using Illumina sequencing for gene discovery and SSR marker identification. PLoS One.

[56] Vijay N., Poelstra J.W., Kunstner A., Wolf J.B. (2013). Challenges and strategies in transcriptome assembly and differential gene expression quantification. A comprehensive in *silico* assessment of RNA-seq experiments. Mol. Ecol..

[57] Trick M., Long Y., Meng J., Bancroft I. (2009). Single nucleotide polymorphism (SNP) discovery in the polyploid *Brassica napus* using Solexa transcriptome sequencing. Plant Biotechnol. J..

[58] Hegedus Z., Zakrzewska A., Agoston V.C., Ordas A., Racz P., Mink M., Spaink H.P., Meijer A.H. (2009). Deep sequencing of the zebrafish transcriptome response to mycobacterium infection. Mol. Immunol..

[59] Wu D., Austin R.S., Zhou S., Brown D. (2013). The root transcriptome for North American ginseng assembled and profiled across seasonal development. BMC Genomics.

[60] Liu Z., Liu P., Luo D., Liu W., Wang Y. (2014). Exploiting Illumina sequencing for the development of 95 novel polymorphic EST-SSR markers in common vetch (*Vicia sativa* subsp. *sativa*). Molecules.

[61] Ono N.N., Britton M.T., Fass J.N., Nicolet C.M., Lin D., Tian L. (2011). Exploring the transcriptome landscape of pomegranate fruit peel for natural product biosynthetic gene and SSR marker discovery. J. Integr. Plant Biol..

[62] Lee M.H., Jeong J.H., Seo J.W., Shin C.G., Kim Y.S., In J.G., Yang D.C., Yi J.S., Choi Y.E. (2004). Enhanced triterpene and phytosterol biosynthesis in *Panax ginseng* overexpressing squalene synthase gene. Plant Cell Physiol..

[63] Sasabe M., Toyoda K., Shiraishi T., Inagaki Y., Ichinose Y. (2002). cDNA cloning and characterization of tobacco ABC transporter: *NtPDR1* is a novel elicitor-responsive gene. FEBS Lett..

[64] Bienert M.D., Siegmund S.E., Drozak A., Trombik T., Bultreys A., Baldwin I.T., Boutry M. (2012). A pleiotropic drug resistance transporter in *Nicotiana tabacum* is involved in defense against the herbivore *Manduca sexta*. Plant J..

[65] Bultreys A., Trombik T., Drozak A., Boutry M. (2009). *Nicotiana plumbaginifolia* plants silenced for the ATP-binding cassette transporter gene *NpPDR1* show increased susceptibility to a group of fungal and oomycete pathogens. Mol. Plant Pathol..

[66] Iseli C., Jongeneel C.V., Bucher P. (1999). ESTScan: A program for detecting, evaluating, and reconstructing potential coding regions in EST sequences. Proc. Int. Conf. Intell. Syst. Mol. Biol..

[67] Fits L.V.D., Memelink J. (2000). ORCA3, a jasmonate-responsive transcriptional regulator of plant primary and secondary metabolism. Science.

